# Big‐Data Analysis of Geometric Descriptors as Efficient Predictors of Energetic Stability in Nonplanar Polycyclic Aromatic Hydrocarbons

**DOI:** 10.1002/jcc.70198

**Published:** 2025-08-01

**Authors:** Kasimir P. Gregory, Amir Karton

**Affiliations:** ^1^ School of Science and Technology University of New England Armidale New South Wales Australia

**Keywords:** CCSD(T), DFT, G4(MP2), isomerization energies, polycyclic aromatic hydrocarbons

## Abstract

Accurate, efficient stability predictors are essential for understanding isomer formation in polycyclic aromatic hydrocarbons (PAHs), with implications for pollution toxicity and carbon‐material design, holding broad environmental and technological significance. Recently, a benchmark study demonstrated that PBE0‐D4 reproduces CCSD(T)‐level isomerization energies for 335 PAHs with a mean absolute deviation (MAD) of 0.67 kcal mol^−1^. Here, we apply the PBE0‐D4/6‐31G(2df,p) level of theory to 38,264 PAH isomers from the COMPAS‐3x database and identify fast, geometry‐based parameters that predict isomer stability. The total dihedral deviation (Σ_Dihedral_) provides a cost‐free nonplanarity metric yielding a mean absolute deviation (MAD) of 3.6 kcal mol^−1^, outperforming maximal *z*‐displacement (MAD = 4.8 kcal mol^−1^) and the Harmonic Oscillator Model of Aromaticity (HOMA; MAD = 5.3 kcal mol^−1^). A combined Σ_Dihedral_–HOMA model reduces the MAD to 2.5 kcal mol^−1^, and adding a fitted semiempirical xTB correction further lowers the MAD to 0.8 kcal mol^−1^. We implement these descriptors in the PAH Automated Property Scanner (PAHAPS) web tool, enabling rapid estimation of PAH isomer energies from molecular coordinates without intensive quantum calculations. This integrated approach facilitates large‐scale screening and efficient design of stable PAH isomers for environmental and materials applications.

## Graphical abstract

Fast, geometry‐only descriptors—Σ_Dihedral_, HOMA, and *θ*_RMSD_—predict isomerization energies for peri‐condensed PAHs. The PAH Automated Property Scanner instantly converts an uploaded *xyz* file into a Δ*E* estimate, enabling large‐scale screening and design.

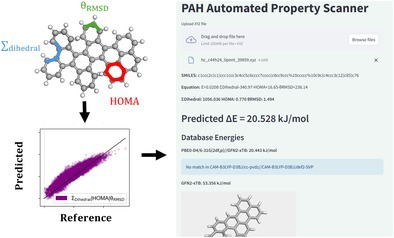

## 1 Introduction

Large chemical databases are transforming the landscape of chemical discovery, enabling researchers to explore vast libraries of chemical structures and properties with unprecedented efficiency. Over the past decade, large DFT databases have emerged, such as the Quantum Machine 9 (QM9) dataset, which includes 134 k equilibrium molecules with up to nine non‐hydrogen atoms [1]. Later, in a tour de force study [2] the atomization energies in the QM9 database were calculated at the CCSD(T) level via the G4(MP2) composite ab initio method [3, 4]. Apart from their important role in training machine‐learning models [5, 6] and benchmarking more approximate theoretical procedures [7, 8], these databases provide powerful means for the systematic search and identification of novel structure–property relationships [9], which are crucial for the design of new materials and molecules in fields such as drug design, materials science, and catalysis. This revolution in chemical discovery is paving the way for more targeted and efficient research.

One of the most ubiquitous classes of organic molecules that are both naturally occurring and synthetic is polycyclic aromatic hydrocarbons (PAHs). As by‐products of incomplete combustion processes—including industrial emissions, vehicle exhaust, and certain cooking methods—PAHs are often characterized as air pollutants with carcinogenic and inflammatory effects [10, 11, 12]. However, they have also found numerous applications in semiconductors [13], energy storage [14, 15], photovoltaics [16], graphene synthesis [17], and pharmaceuticals [18] as well as other aspects of organic and materials chemistry [16, 19, 20, 21, 22, 23]. The relative stability of PAH isomers is an important factor in understanding their chemical reactivity and potential applications. For instance, PAHs have been explored as components in the synthesis of graphene‐like materials, which are known for their exceptional mechanical strength and electrical conductivity [24]. Another important area is energy storage applications, such as for durable anode materials in sodium‐ion batteries [25]. The ability to predict the relative stability of PAH isomers using computationally economical methods has attracted considerable attention in recent years. This can be achieved through direct energetic calculations [26, 27, 28, 29, 30], by evaluating other chemical properties such as aromaticity [31, 32], or by analyzing structural parameters that correlate with energetic stability [33, 34, 35, 36, 37]. Since 2022, the Gershoni‐Poranne group has been developing an extensive database of PAHs known as the COMPAS database (COMputational database of Polycyclic Aromatic Systems) [26, 27, 28]. This database has thus far undergone four phases of development: cata‐condensed polybenzenoid hydrocarbons (phase 1, 34 k structures) [26], cata‐condensed hetero‐polycyclic aromatic systems (phase 2, 500 k structures) [27], peri‐condensed heterocyclic aromatic systems (phase 3, 39 k structures) [28], and boron‐nitrogen pair substituted cata‐condensed polybenzenoid hydrocarbons (phase 4, 24 k structures) [38]. The COMPAS database provides a valuable resource for exploring PAH structure–property relationships and serves as a benchmark for validating computational methodologies, particularly in predicting key properties such as ground‐state energies.

We recently obtained accurate CCSD(T) isomerization energies for a set of 335 PAHs (a.k.a. the PAH335 database) with up to 36 carbon atoms using the G4(MP2) thermochemical protocol [29]. The G4(MP2) composite ab initio method [3, 4] approximates the CCSD(T) energy (i.e., coupled cluster with singles, doubles, and quasiperturbative triple excitations) in conjunction with a triple‐ζ‐quality basis set. It has been shown to provide excellent hydrocarbon isomerization energies [39, 40, 41, 42], with mean absolute deviations (MADs) below the threshold of “chemical accuracy” (arbitrarily defined as ~1 kcal mol^−1^) [43]. The PAH335 database includes PAH isomers ranging from C_17_H_12_ to C_36_H_16_, including planar and nonplanar structures with 4–11 condensed rings. As such, this database is structurally diverse and provides an excellent means for identifying DFT methods that can accurately reproduce the G4(MP2) isomerization energies. We find that the hybrid generalized gradient approximation (HGGA) functional PBE0‐D4 [44, 45] attained a remarkably low MAD of only 0.67 kcal mol^−1^ from the G4(MP2) reference data. It should be noted that the hybrid‐meta GGA (HMGGA) functionals TPSSh‐D4 [46] and PW6B95‐D4 [47] were close seconds with a MAD of 0.72 kcal mol^−1^. We also note that the SVWN5 [48] local density approximation (LDA) functional performs surprisingly well with a MAD of 0.91 kcal mol^−1^. Since the computational cost of HGGA and HMGGA methods is much higher than that of LDA methods, this latter result could prove useful for cases where computational cost is a limiting factor.

The isomerization energies in the COMPAS‐3x database have been calculated at the CAM‐B3LYP‐D3BJ/aug‐cc‐pVDZ level of theory [28, 49, 50]. The CAM‐B3LYP method attains a MAD of 1.70 kcal mol^−1^ relative to the G4(MP2) reference values in the PAH335 database [29]. Inclusion of the empirical D4 dispersion correction slightly increases the MAD to 1.99 kcal mol^−1^. The PBE0‐D4 functional cuts this MAD by 66% and attains a value of MAD merely 0.67 kcal mol^−1^ relative to the G4(MP2) reference values. In the present work, we use the PBE0‐D4 functional in conjunction with the triple‐ζ‐quality 6‐31G(2df,p) Pople‐style basis set to calculate the isomerization energies of the ~40 k peri‐condensed PAHs in the COMPAS‐3x database [28]. Herein, we use this extensive set of isomerization energies to identify structural parameters that correlate with energetic stability and can serve as energetic predictors. We aim to identify a priori descriptors that can reliably predict the stability of PAH isomers with minimal computational overhead. We provide an online tool to calculate these descriptors and approximate (or find) the isomerization energies of PAHs from its *xyz* structure.

## 2 Theory and Computational Methods

All equilibrium structures were taken from the COMPAS‐3x database [28], which used GFN2‐xTB calculations (hereinafter referred to as XTB) to optimize all geometries [51, 52]. All the DFT single point energy calculations were carried out at the PBE0‐D4/6‐31G(2df,p) level of theory [44, 45, 53, 54, 55, 56, 57, 58] using the Gaussian 16 program suite [59]. The isomerization energies, $\Delta E_{\mathrm{iso}}$, are calculated relative to the minimum energy isomer at that level of theory such that $\Delta E_{\mathrm{iso}}=E_{\mathrm{iso}}-E_{\min}$. All Python analyses scripts, using SciPy [60], RDKit, SciKit‐learn [61], NumPy [62] and Pandas are contained in the Supporting Information or may be found on GitHub (https://github.com/Kasigee/PAH_Analysis). These scripts and the PAH webtool (https://pahaps.streamlit.app/) were developed with the aid of ChatGPT (4o, o3 and o4‐mini high). For geometric parameter fits to the isomerization energies, we use weighted linear regression to acquire a linearized form, for example:

(1) $$\begin{array}{c} \Delta E_{\mathrm{iso}}=a_{0}+a_{1}\sum_{\text{Dihedral}}+a_{2}\text{HOMA}+\ldots \end{array}$$

where the empirical parameters (*a*_*n*_) are optimized to minimize the MAD relative to the PBE0‐D4 reference values. This approach provides a baseline for understanding the individual contribution of each variable to the target prediction, which enables the identification of synergistic effects between variables for improved prediction accuracy.

## 3 Results and Discussion

### 3.1 PBE0‐D4/6‐31G(2df,p) Benchmark on COMPAS‐3x Isomers

Identifying reliable structure‐energy relationships requires examining a sufficiently large set of isomers that capture a wide range of structural variations and energy landscapes. Therefore, we consider only subsets of COMPAS‐3x, which include over 1000 isomers in each subset. The subsets considered in the present work are listed in Table 1. Overall, these subsets include 38,264 isomers with 9–11 aromatic rings. For brevity, the 38,264 isomers considered here will be referred to as the COMPAS‐3x’ database. We note that these isomers comprise 97% of the 39,482 isomers in the entire COMPAS‐3x database. Thus, excluding subsets with less than 1000 isomers is not expected to significantly affect the diversity of the COMPAS‐3x’ subset considered here. This comprehensive dataset allows for the detection of subtle patterns and correlations between molecular geometry and energetic stability. All PBE0‐D4/6‐31G(2df,p) isomerization energies (∆*E*_iso_) and investigated parameters are provided in Table S1 of the Supporting Information.

**TABLE 1:** Overview of the isomer subsets in the COMPAS‐3x’ database considered in the present work. Only subsets with more than 1000 isomers are considered here, totaling 38,264 isomers comprising of 9–11 rings. The average, smallest, and largest isomerization energies (∆*E*_iso_) obtained at the PBE0‐D4/6‐31G(2df,p) level of theory are given in kcal mol^−1^.

Subset	Number of isomers	Average ∆*E*_iso_	Smallest ∆*E*_iso_	Largest ∆*E*_iso_
C_36_H_20_	1182	15.00	0.21	37.16
C_38_H_20_	1594	15.00	0.64	38.74
C_40_H_20_	1683	17.60	1.07	43.27
C_40_H_22_	5084	17.23	0.20	42.76
C_42_H_22_	7662	16.77	0.24	42.20
C_44_H_24_	21,059	18.75	0.47	54.74
All	38,264	17.82	0.20	54.74

### 3.2 Distribution of Isomerization Energies and Planarity

**FIGURE 1:**
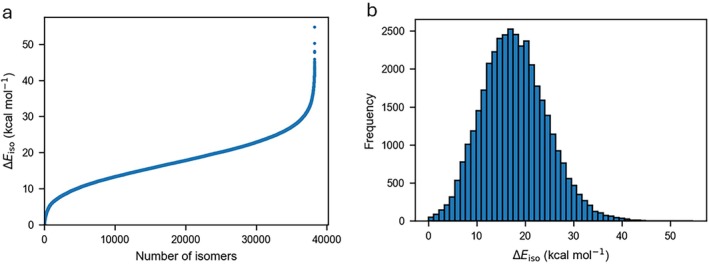
(a) Overview of the PBE0‐D4/6‐31G(2df,p) isomerization energies for 38,264 isomers in the COMPAS‐3x’ database in kcal mol^−1^. (b) Histogram of the distribution of the relative isomerization energy.

Before proceeding to analyze structure‐energy relationships in the COMPAS‐3x’ database, it is instructive to examine the PBE0‐D4/6‐31G(2df,p) isomerization energies relative to the most energetically stable structure within each isomer subset. Figure 1a plots the PBE0‐D4/6‐31G(2df,p) isomerization energies for the 38,264 structures in the database. The PBE0‐D4/6‐31G(2df,p) isomerization energies for all isomers are given in the Supporting Information. The isomerization energies vary smoothly up to 45.9 kcal mol^−1^ (Figure 1). As observed for energy distributions of other isomers [63, 64], the energy distribution in Figure 1 exhibits a logit‐type distribution with larger variations in the isomerization energies of the most and least stable isomers and a flatter region in between with smaller energy variations. The histogram in Figure 1b shows a right‐skewed energy distribution. For example, the 750 most stable isomers on the left (i.e., 2% of the isomers) span a relatively narrow energetic range between 0.0 and 5.4 kcal mol^−1^, whereas the 750 least stable isomers on the right span a much wider energetic range between 32.7 and 54.8 kcal mol^−1^. The remaining 36,759 isomers (i.e., 96% of isomers) are associated with relative energies between 5.8 and 32.7 kcal mol^−1^. The average isomerization energy over the entire set is 17.8 kcal mol^−1^, 52% of the isomers are associated with relative energies below the average isomerization energy, and 48% of isomers are associated with relative energies above the average isomerization energy. Table 1 gives the average isomerization energy for the individual isomer subsets. Generally speaking, the average isomerization energy increases with the size of the PAH. For example, for the isomers with 36 and 38 carbon atoms, it is 15.0 kcal mol^−1^, whereas for the isomers with 40–44 carbon atoms, it ranges between 16.8 and 18.8 kcal mol^−1^ (Table 1). Similarly, the largest isomerization energy in each subset tends to increase with the size of the PAH. The largest isomerization energy in each of the subsets is 37.16 (C_36_H_20_), 38.74 (C_38_H_20_), 43.27 (C_40_H_20_), 42.76 (C_40_H_22_), 42.20 (C_42_H_22_), and 54.74 (C_44_H_24_) kcal mol^−1^.

**FIGURE 2:**
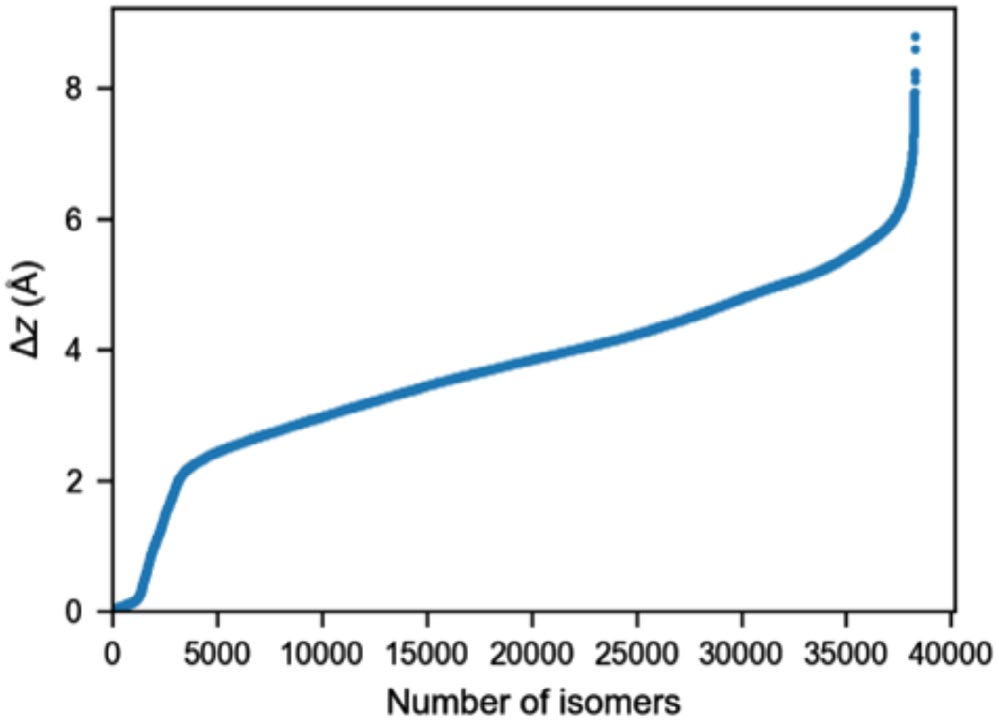
Overview of the deviation from planarity as measured by ∆*z* values for 38,264 isomers in the COMPAS‐3x’ database in Å.

**FIGURE 3:**
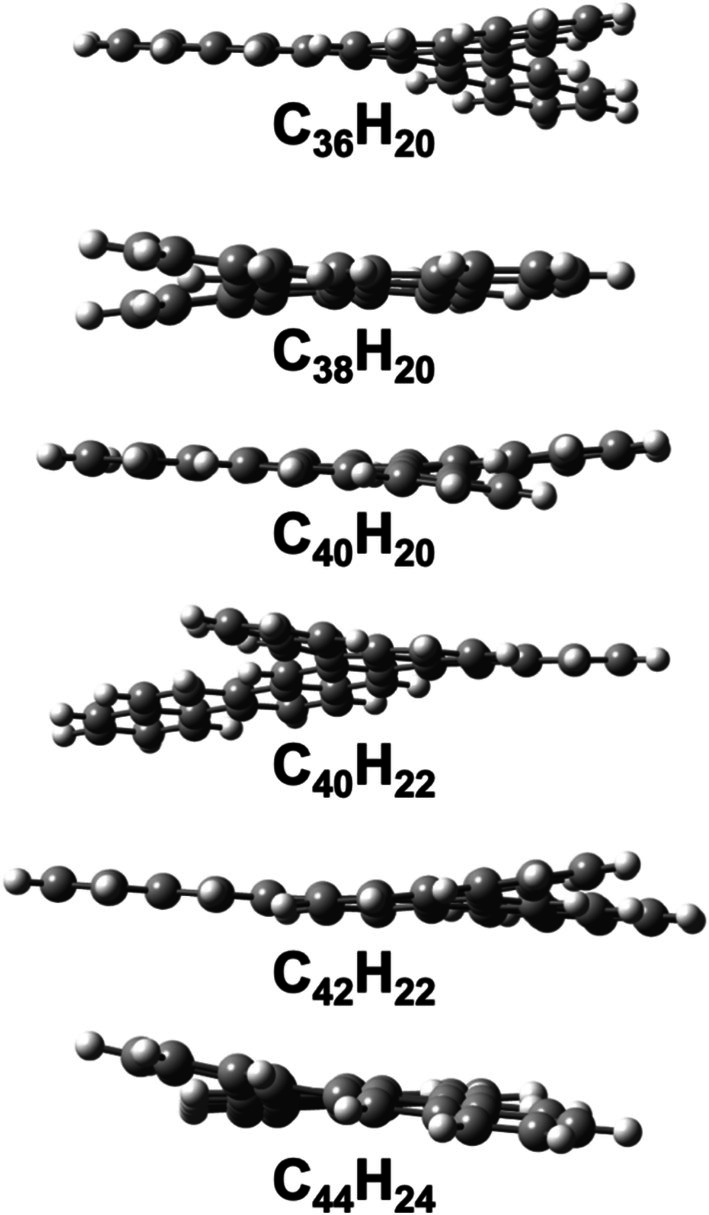
Isomers with a ∆*z* value of 1.0 Å from each subset in the COMPAS‐3x’ database.

Wahab and Gershoni‐Poranne have found a general correlation between the isomerization energy and the number of helical motifs present in the PAH, indicating that nonplanarity plays a significant role in the relative energies [28]. They also suggested that the deviation from planarity of the PAHs can be measured by placing the PAH on the *xy*‐plane and taking the difference between the highest and lowest coordinates on the *z*‐axis (this distance is denoted as ∆*z*). Figure 2 plots the ∆*z* values for the 38,264 isomers examined here. The ∆*z* values range from 0.01 to 8.78 Å. Figure 2 can be divided into four fairly linear regions: a flat region with ∆*z* values between 0.0 and 0.2 Å, which can be considered clearly planar. There are ~1200 isomers in this region (i.e., ~3% of isomers). For the next set of ~2000 (or ~5% of) isomers, the ∆*z* values rise sharply between ∆*z* = 0.2–2 Å. It should be noted that isomers with a ∆*z* value of 1 Å are already visibly nonplanar. To illustrate this point, Figure 3 depicts five isomers with ∆*z* = 1.0 Å, one from each subset (C_36_H_20_, C_38_H_20_, C_40_H_20_, C_40_H_22_, C_42_H_22_, C_44_H_24_). For most isomers (i.e., ~34,000 isomers or ~90%), the ∆*z* values steadily increase from 2 to 6 Å. Finally, ~1000 isomers are highly nonplanar with ∆*z* values of 6–9 Å. The above results illustrate that most of the 38,264 isomers considered here are associated with moderate to high degrees of nonplanarity. For example, 92% of isomers have ∆*z* values between 2.0 and 8.8 Å. It should also be noted that Wahab and Gershoni‐Poranne [28] observed reasonably large deviations in Δ*z* were common between XTB and DFT optimized structures near planarity. This could result in significant differences in energies predicted by these models from geometric predictors of the same isomer.

It is of interest to examine the performance of the XTB semiempirical method for the planar and nonplanar isomers considered here relative to the PBE0‐D4/6‐31G(2df,p) isomerization energies. Table 2 lists these error statistics. Remarkably, the XTB method attains almost three times better accuracy for the 1978 planar (or nearly planar) isomers with ∆z values smaller or equal to 1.0 Å than the nonplanar isomers with ∆z values greater than 1.0 Å. In particular, for the planar systems, XTB attains MAD, RMSD, and maximum deviation values of 2.07, 2.37, and 6.58 kcal mol^−1^, respectively. In contrast, for nonplanar systems, these values increase to 6.08, 6.53, and 16.51 kcal mol^−1^, respectively. This highlights the challenges faced by XTB in accurately modeling the energetics of nonplanar systems, which comprise the majority of the COMPAS3x database. Introducing a correction factor to XTB from the line of best fit (C.XTB, Table S2) reduces the MAD; however, this reduces its transferability.

**TABLE 2:** Error statistics for the local XTB method and PBE0/6‐31G(2df,p) for the planar and nonplanar isomers considered here relative to the PBE0‐D4/6‐31G(2df,p) isomerization energies (in kcal mol^−1^).

	Subset	MAD	RMSD	MSD	Max Dev
XTB	Planar (1978)^a^	2.07	2.37	−2.06	6.58
	Nonplanar (36,286)^b^	6.08	6.53	−6.08	16.51
	Everything (38,264)	5.87	6.38	−5.87	16.51
PBE0	Planar (1978)^a^	0.86	0.99	−0.82	2.25
	Nonplanar (36,286)^b^	2.59	3.76	2.53	24.24
	Everything (38,264)	2.50	3.66	2.35	24.24

Abbreviations: MAD = mean absolute deviation; Max Dev = maximum deviation; MSD = mean signed deviation; RMSD = root‐mean‐square deviation.

^a^ Planar or nearly planar isomers with ∆*z* values smaller or equal to 1.0 Å.

^b^ Nonplanar isomers with ∆*z* values larger than 1.0 Å.

We also investigated the effects of D4 dispersion on isomerization energies by excluding it (i.e., PBE0/6‐31G(2df,p)) and comparing the performance between planar and nonplanar systems. Without dispersion, PBE0 shows superior accuracy overall and significantly greater transferability than XTB. For planar systems, PBE0 achieves a MAD of 0.86 kcal mol^−1^, while for nonplanar systems, the MAD increases to 2.59 kcal mol^−1^. By comparison, XTB's MAD increases from 2.1 to 6.1 kcal mol^−1^ when moving from planar to nonplanar systems.

### 3.3 Performance of Geometric Descriptors

Given the prohibitive computational cost of quantum chemical methods like PBE0, especially for large PAHs, it is crucial to explore fast structural parameters as predictors for properties such as the isomerization energy. These parameters offer a computationally efficient alternative for studying large systems without relying on high‐performance computing resources.

Aromaticity has become a popular descriptor for the stability of PAHs since Clar's sextet rule was introduced in 1972 [33], leading to a variety of methods for its approximation. Since then, many geometric, topological, and electronic indices have been developed to approximate aromaticity and other stability‐related properties. For example, Nguyen and Truong [34] proposed the degree of π–orbital overlap based on a two‐dimensional structural representation of a PAH to describe the aromaticity in a simpler way than something like the BG (1) descriptor [65] of Clar's theory. Similarly, Fite et al. [66] used text‐based descriptors as unique structural identifiers for machine learning models structure–property relationships, while Weiss et al. [67] extended this idea by incorporating a graph‐of‐rings representation. Both Fite et al. [66] and Weiss et al. [66] identified and utilized cove, fjord, and helix motifs—which effectively represent increasing dihedral angles—as a means to determine isomerization energies. These models have demonstrated remarkable accuracy, predicting HOMO‐LUMO gaps and relative energies with MADs as low as 0.25 ± 0.09 and 0.12 ± 0.05 kcal mol^−1^, respectively. These latter three methods were formulated for cata‐condensed PAHs and are therefore more applicable to datasets like COMPAS‐1 [26].

The nucleus independent chemical shift (NICS) is another widely used aromaticity descriptor, and correlates well with HOMO‐LUMO gaps and ionization potentials [68]. Predi‐XY is a fast, efficient method for predicting these based on precomputed building blocks [69]. It is currently made for cata‐condensed systems, so it lacks the peri‐condensed building blocks to describe the PAHs in COMPAS‐3. Given that explicit calculations of full NICS profiles are computationally prohibitive, these have not been explored here given the size of the database.

Among the commonly used geometric indices is the Harmonic Oscillator Model of Aromaticity (HOMA), which is a metric to quantify π–electron delocalization in aromatic systems. The HOMA index evaluates bond‐length deviations from an optimal aromatic value using the formula:

(2) $$\text{HOMA}=1-\frac{\alpha }{n}\sum_{k=1}^{n}{\left(R_{k}-R_{\mathrm{opt}}\right)}^{2}$$

where the bond lengths within each ring, *R*_*k*_, are compared to an optimal bond length, *R*_opt_ (typically 1.388 Å for aromatic C–C bonds) [70], *n* is the number of bonds in the ring and *α* is a normalizing constant (set to 257.7 in this case for C–C). HOMA values closer to 1 indicate higher aromaticity as they correspond to bond lengths approaching the optimal value.

In this study, we computed the average HOMA across all rings and tested several values of *R*_opt_, including 1.3914 Å (the CCSD(T)/cc‐pVQZ optimized C–C bond distance in benzene of from Gauss and Stanton) [71] and 1.397 Å (the NIST listed experimental value from Herzberg) [72]. Additionally, we explored a broader range (0–5 Å) to investigate the sensitivity of the HOMA index and identify optimal values for predicting isomer stability. We also evaluated two modified versions of the HOMA index: HOMA_fused_, which only considered C–C bonds between rings, and HOMA_edge_, which only considered C–C bonds along the PAH perimeter. In all cases, HOMA_fused_ outperformed HOMA_edge_, so error analyses for HOMA_edge_ are not reported here. Full methodological details are provided in the Supporting Information. Notably, HOMA has been stated to have issues with large pericondensed PAHs due to the local aromaticity of adjacent rings [73]. A related metric is the bond length alternation (BLA), which assesses uniformity of bond lengths within a ring, with “perfect” aromaticity characterized by equal bond lengths throughout the ring [74].

The aromatic parameters listed have limitations on being a universal parameter for systems where nonplanarity occurs, as they rely on 2D structural representations of the PAH, which will struggle to capture nonplanarity and cannot capture conformer effects (e.g., bent vs. helical configurations of nonplanar structures). Consequently, they may be prone to “conformational noise” in any fits between these data and the representations. In contrast, many 3D geometric parameters inherently account for conformational effects and are better suited for analyzing more complex PAHs.

To complement HOMA and address its limitations, we evaluated additional structural parameters that can be computed rapidly and efficiently (Table 3). These include:

- Bond‐length metrics: Average C–C bond lengths (*l*_avg_), BLA.
- Bond‐angle metrics: The sum and RMSD from the ideal 120° bond angle (Σ*θ* and *θ*_RMSD_, respectively) and bond angle alternation (BAA). Provide insights into the extent of strain and distortion within the molecular structure, impacting aromaticity and overall stability.
- Nonplanarity metrics: Sum of the C–C–C–C dihedral angle deviations from 0° or 180° (Σ_Dihedral_), corresponding to cis and trans configurations, respectively, the RMSD of the pyramidalization angle (*θ*_pyr‐RMSD_), which quantifies how much an sp^2^‐hybridized carbon atom deviates from the plane defined by its three bonded atoms, and maximum *z*‐displacement (Δ*z*) which is the magnitude of the maximum and minimum *z* values of the structure (when the molecules were placed in the *xy* plane).

**TABLE 3:** Error statistics for various fitted structural parameters method and variants thereof for all (A) 38,264 isomers considered here, and the 1978 planar (P) and 36,286 nonplanar (N) subsets, relative to the PBE0‐D4/6‐31G(2df,p) isomerization energies (in kcal mol^−1^).

Subset	MAD			RMSD			MaxD		
	A	P	N	A	P	N	A	P	N
HOMA	5.30	2.43	5.06	6.66	3.10	6.36	36.62	12.47	35.75
HOMA_fused_	5.31	2.09	5.08	6.66	2.72	6.38	36.83	12.29	36.19
BLA	5.19	2.98	4.95	6.52	3.71	6.23	36.80	13.91	36.26
*l*_avg_	4.91	3.13	4.73	6.13	3.80	5.92	28.57	12.03	28.44
BAA	5.00	3.21	4.95	6.31	3.90	6.25	37.23	12.57	36.79
Σ*θ*	4.89	3.21	4.67	6.15	3.91	5.87	29.03	12.56	28.46
*θ*_RMSD_	4.58	3.14	4.30	5.79	3.82	5.39	24.91	13.53	23.93
∆*z*	4.78	3.20	4.84	6.03	3.90	6.09	37.77	12.42	37.48
Σ_Dihedral_	3.62	3.21	3.58	4.47	3.91	4.43	23.20	12.43	21.94
*θ*_pyr‐RMSD_	4.33	3.20	4.37	5.32	3.90	5.35	28.82	12.36	28.00
Σ_Dihedral_|HOMA	2.53	2.43	2.42	3.23	3.10	3.09	16.48	12.60	15.23
Σ_Dihedral_|HOMA_fused_	2.44	2.08	2.30	3.12	2.73	2.93	18.18	12.27	16.41
Σ_Dihedral_|*θ*_RMSD_	3.42	3.12	3.40	4.22	3.80	4.20	19.10	13.33	18.57
HOMA|*θ*_RMSD_	4.58	2.26	4.26	5.78	2.86	5.35	24.80	11.17	23.58
Σ_Dihedral_|HOMA|*θ*_RMSD_	2.48	2.22	2.39	3.18	2.83	3.07	16.09	11.17	14.93
Σ_Dihedral_|HOMA_fused_|*θ*_RMSD_	2.30	1.98	2.21	2.96	2.54	2.83	16.47	11.09	15.08
C.XTB	1.16	0.46	1.16	1.54	0.57	1.54	10.37	1.77	10.11
*θ*_RMSD_|C.XTB	1.15	0.45	1.14	1.53	0.55	1.51	10.09	1.77	9.65
HOMA|C.XTB	1.09	0.37	1.12	1.48	0.48	1.48	10.06	2.41	9.95
Σ_Dihedral_|C.XTB	0.88	0.45	0.88	1.16	0.56	1.16	7.80	1.71	7.48
Σ_Dihedral_|HOMA|C.XTB	0.82	0.36	0.80	1.08	0.46	1.05	6.84	2.29	6.18
Σ_Dihedral_|HOMA|*θ*_RMSD_|C.XTB	0.82	0.32	0.80	1.08	0.40	1.05	6.82	2.22	6.29
Σ_Dihedral_|HOMA_fused_|*θ*_RMSD_|C.XTB	0.84	0.30	0.82	1.10	0.39	1.08	7.14	2.49	6.51

Abbreviations: ∆*z* = maximum z‐displacement; BAA = bond angle alternation; BLA = bond length alternation; C.XTB = corrected XTB, from a line of best fit; HOMA = Harmonic Oscillator Model of Aromaticity; HOMA_fused_ = HOMA on only fused C–C bonds between aromatic rings; *l*_avg_ = mean C–C bond length; MAD = mean absolute deviation; MaxD = maximum deviation; MSD = mean signed deviation; RMSD = root‐mean‐square deviation; *θ*_pyr—RMSD_ = the RMSD of the pyramidalization angle; *θ*_RMSD_ = RMSD of bond angles from 120°; Σ_Dihedral_ = sum of dihedral angles away from 0°/180°; Σθ = sum of bond angle deviations from 120°.

Using a weighted linear regression model, among these, Σ_Dihedral_ proved to be the strongest predictor of isomerization energy, achieving a MAD of 3.62 kcal mol^−1^ when fitted to PBE0‐D4/6‐31G(2df,p) data. This metric offers a comprehensive assessment of nonplanarity by capturing distortions distributed across the entire molecular structure. We note that, Δ*z*, which focuses on a single distance across the molecular structure, results in a MAD of 4.78 kcal mol^−1^. Similarly, the RMSD of pyramidalization angles showed moderate performance (MAD: 4.33 kcal mol^−1^). None of the lone bond‐length or bond‐angle metrics were as good a predictor overall; however, HOMA_fused_ was the best‐performing structural predictor tested for the planar systems (MAD: 2.09 kcal mol^−1^). Due to the planar systems generally being the most stable isomers, a MAD of 3.21 kcal mol^−1^ indicates no predictive power for this subset. We also investigated the combination of these geometric parameters using a weighted linear regression approach. Notably, combining Σ_Dihedral_ and HOMA significantly improved predictions, reducing the MAD to 2.53 kcal mol^−1^. Adding *θ*_RMSD_ provided only minor additional improvements to the current dataset, with a MAD of 2.48 kcal mol^−1^. HOMA_fused_ showed slight advantages over HOMA, except when used in combination with XTB.

We also assessed whether these geometry parameters could be used to predict HOMO–LUMO band gaps. Overall, the correlations were weak. Most individual parameters had *R* ^2^ values below 0.1, with MADs around 0.3 eV. Among them, HOMA_fused_ performed best, with an *R* ^2^ of 0.33 and MAD of 0.24 eV. The best combined models achieved *R* ^2^ of approximately 0.55 and an MAD near 0.2 eV—substantially higher than the 0.005 eV MADs reported for predictive models of catacondensed PAHs [67].

**FIGURE 4:**
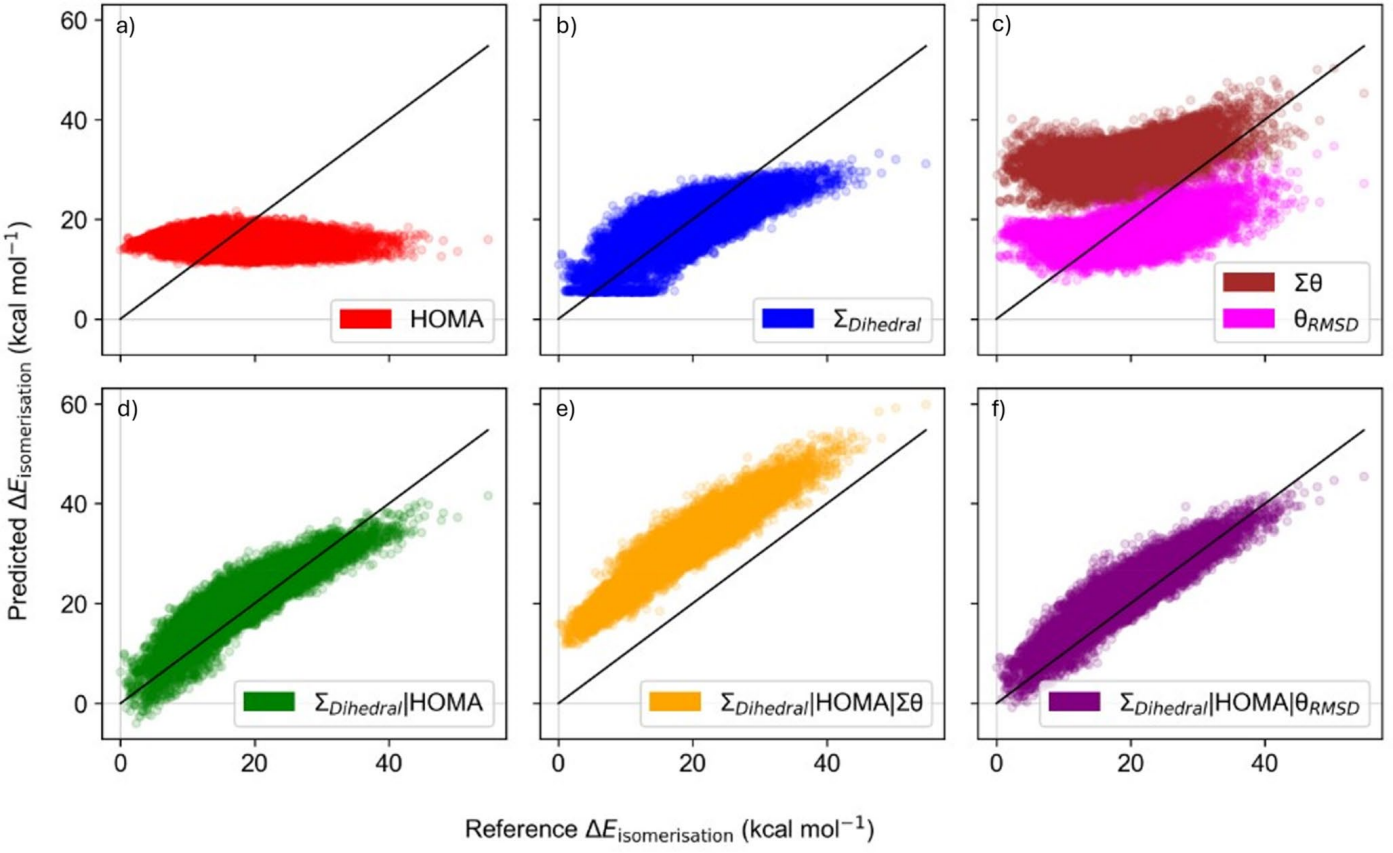
Comparison of the single‐parameter (a) HOMA, (b) Σ_Dihedral_ and (c) Σ*θ* or *θ*_RMSD_, as well as the combined (d) Σ_Dihedral_|HOMA, (e) Σ_Dihedral_|HOMA|Σ*θ*, and (f) Σ_Dihedral_|HOMA|*θ*_RMSD_ fits used to predict the PBE0‐D4/6‐31G(2df,p) isomerization energies of C_44_H_24_. All fits were trained on the smaller C_36_H_20_ dataset.

Figure 4 compares four different structural descriptors, HOMA, the Σ_Dihedral_, Σ*θ* or *θ*_RMSD_—used individually and in combination—for predicting the PBE0‐D4/6‐31G(2df,p) isomerization energies. Each descriptor (or descriptor set) was fitted to optimize (i.e., its slope(s) and intercept determined for Equation 1) against the smaller C_36_H_20_ isomer set (1182 geometries), then applied to the larger C_44_H_24_ isomer set (21,059 geometries). In isolation, the HOMA parameter (Figure 4a) shows no predictive power for the C_44_H_24_ isomerization energy, where the fit predicts every structure clustered between 10 and 20 kcal mol^−1^, even though the actual reference energies range between 0 and 55 kcal mol^−1^. Σ_Dihedral_ (Figure 4b) shows some predictive power with the overall trend matching the reference energies. However, planar PAHs have Σ_Dihedral_ ~ 0, which introduces an offset of a few kcal mol^−1^ as the model tries to fit both planar and highly distorted isomers into a single linear relationship. There is also a nonlinearity to the fit where high isomerization energies are not sufficiently captured by the linear fit to Σ_Dihedral_. Notably, when investigating parameters based on the bond angle, the Σ*θ* parameter is shifted from the primary trend (Figure 4c), while θ_RMSD_ does not. This likely reflects the increasing number of rings when moving from the smaller to the larger system: Σ*θ* grows simply by counting more angles, whereas *θ*_RMSD_ is inherently normalized by looking at average deviations. In contrast, Σ_Dihedral_ remains aligned with the overall correlation, implying that nonplanarity is effectively a global parameter in these systems. Although HOMA (or HOMA_fused_) alone is ineffective in capturing nonplanar PAH isomerization energies in isolation, when paired with Σ_Dihedral_ a synergy arises between the two parameters (Figure 4d). HOMA seems to allow for differentiation of the planar PAHs such that the fit reaches some predicted Δ*E*_isomerization_ values around 0, and the nonlinearity improves, mitigating the limitations of Σ_Dihedral_ in isolation. When incorporating bond angle parameters (Figure 4e,f) we note that the linearity of the fit improves further, and *θ*_RMSD_ is a more transferable angle parameter than Σ*θ* between PAHs of varied numbers of rings, as was the case in Figure 4c.

Four isomers show notably higher isomerization energies (47.8–54.8 kcal mol^−1^), as evident in Figure 1. These structures are characterized by significant nonplanarity and highly connected rings; the latter is particularly prominent. If we quantify highly connected rings by counting any structural node, whether an individual benzene ring or entire peri‐condensed fragment, where three or more fused ring connections intersect, each of these isomers exhibits approximately 4–5 such rings. Although discrete values like these are less valuable as standalone fitting parameters, especially in large datasets where thousands of isomers may share the same value, they can still be informative when used alongside other descriptors.

**FIGURE 5:**
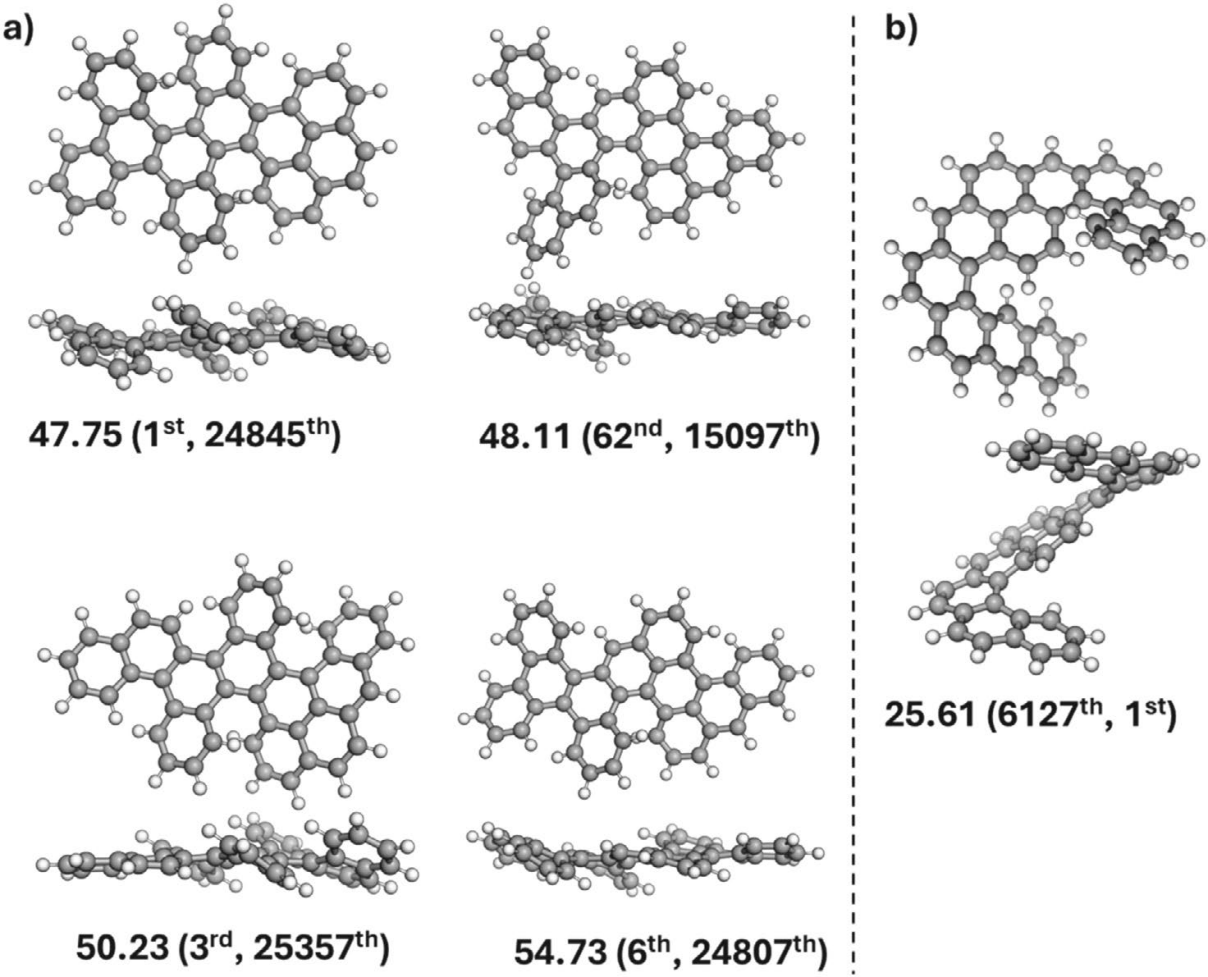
(a) The four highest PBE0‐D4/6‐31G(2df,p) isomerization energies (in kcal mol^−1^) from the COMPAS‐3x’ database. (b) The isomer exhibiting the largest out‐of‐plane distortion (highest Δ*z*), which has an isomerization energy less than half the highest‐energy isomer. All isomers belong to the C_44_H_24_ subset. The ranks for Σ_Dihedral_ (left) and Δ*z* (right) are given in parentheses, with first denoting the largest value of that parameter across the dataset.

The highest‐energy structure in this dataset illustrates the relative utility of Σ_Dihedral_ versus Δ*z* (see Figure 5). This isomer displays a high degree of local nonplanarity. Its Σ_Dihedral_ of 1770° is the sixth largest in the dataset, whereas the maximum is 1912°. However, because these dihedral distortions are spread throughout the molecule, the Δ*z* remains relatively small (3.30 Å, the 24807th largest of the dataset), suggesting only minor overall nonplanarity. In contrast, the isomer with the largest Δz of 8.78 Å (Figure 5b) has an isomerization energy roughly half that of the highest‐energy structure (25.6 vs. 54.73 kcal mol^−1^). It consists of three planar fragments (each locally stable) that are tilted relative to one another, forming a helical arrangement. Branching does not significantly contribute to destabilization in that structure (apart from a small pericondensed section), so its Σ_Dihedral_ is only 1178°, about half of the dataset's maximum. Despite its large global distortion (Δ*z*), this isomer ranks 4721st in isomerization energy and 6127th in Σ_Dihedral_. This, in conjunction with the highest isomerization energy structure's high dihedral rank, underscores how Σ_Dihedral_ can better capture distributed nonplanarity than Δ*z*. These outliers highlight that distributed local distortions captured by Σ_Dihedral_ matter more than a single large amplitude distortion measured by Δ*z* in the relative stability of these PAH isomers. These qualitative observations corroborate the quantitative trends observed in Table 3.

### 3.4 Transferability Across PAHs of Various Sizes

**FIGURE 6:**
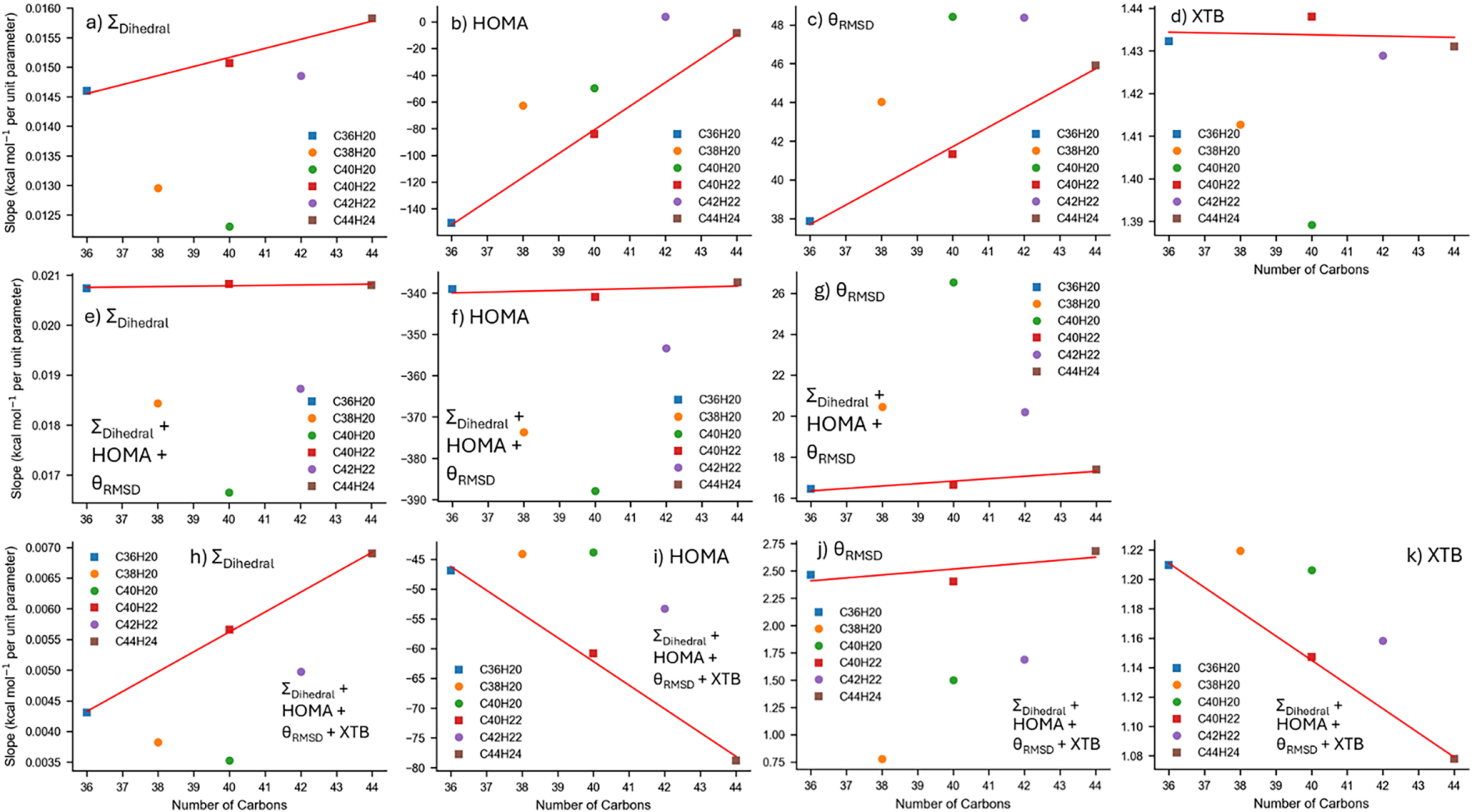
Plots of slope from fitted relationship to the PBE0‐D4/6‐31G(2df,p) reference isomerization energies (in kcal mol^−1^) versus the number of carbons for six different approaches: The (a) Σ_Dihedral_, (b) HOMA, (c) *θ*_RMSD_, and (d) XTB from their lone fits, the (e) Σ_Dihedral_, (f) HOMA and (g) *θ*_RMSD_, from the Σ_Dihedral_, HOMA and *θ*_RMSD_ combined fit and the (h) Σ_Dihedral_, (i) HOMA, (j) *θ*_RMSD_ and (k) XTB from the Σ_Dihedral_, HOMA, *θ*_RMSD_ and XTB combined fit. Each marker represents a different chemical formula, with squares denoting the C_16+4*n*_H_10+2*n*_ series (i.e., C_36_H_20_, C_40_H_22_, C_44_H_24_) and circles denoting other formulas. Red lines show linear fits to the square‐labeled data.

To investigate the transferability and extrapolation of parameter relationships across different system sizes, we analyzed how the fitted slopes and intercepts varied between chemical formulas (Figures 6 and S1–S4). This approach provides valuable insights into whether trends observed in one system could be reliably extended to others, a key consideration for predicting properties and behaviors in structurally diverse molecules. For the structures corresponding to the chemical formula series C_16+4*n*_H_10+2*n*_ (e.g., C_36_H_20_, C_40_H_22_, C_44_H_24_), we observed a distinct trend in their slopes and intercepts, as well as in the errors of the fits. This consistent deviation suggests that the C_16+4*n*_H_10+2*n*_ series shares structural functionality that influences the relationship between these parameters and isomerization energies in a distinct manner. While the MADs and maximum errors for this series are often worse compared to other systems (Figures S5 and S6, respectively), the consistency in trends highlights their potential for improved extrapolation and transferability. These trends were slightly displaced from those seen in another set of chemical formulas, specifically C_38_H_20_, C_40_H_20_, and C_42_H_22_.

**FIGURE 7:**
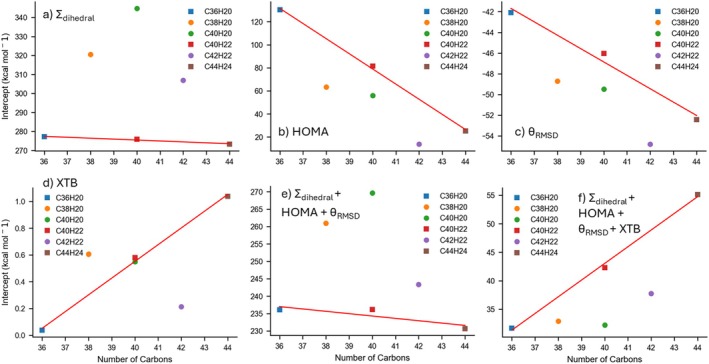
Plots of *y*‐intercept from fitted relationship to the PBE0‐D4/6‐31G(2df,p) reference isomerization energies (in kcal mol^−1^) versus the number of carbons for six different approaches: The (a) Σ_Dihedral_, (b) HOMA, (c) *θ*_RMSD_, (d) XTB from their lone fits, the (e) Σ_Dihedral_, HOMA and θ_RMSD_ combined fit, and the (f) Σ_Dihedral_, HOMA, *θ*_RMSD_ and XTB combined fit. Each marker represents a different chemical formula, with squares denoting the C_16+4*n*_H_10+2*n*_ series (i.e., C_36_H_20_, C_40_H_22_, C_44_H_24_) and circles denoting other formulas. Red lines show linear fits to the square‐labeled data.

The transferability of trends in the C_16+4*n*_H_10+2*n*_ series is further supported by the consistent slopes and intercepts observed for certain parameter combinations. This is most prominent when analyzing the combination of Σ_Dihedral_, HOMA, and *θ*_RMSD_ parameters. For these three parameters, the maximum deviations in each respective slope across the C_16+4*n*_H_10+2*n*_ series were limited to 0.5%, 1.1%, and 5.5%, respectively (Figure 6e–g). The intercept for this model (Figure 7e) across the C_16+4*n*_H_10+2*n*_ series only deviated by as much as 2.5% (236.1, 236.1 and 230.7 for C_38_H_20_, C_40_H_20_, and C_42_H_22_, respectively). These findings underline the potential of these parameters to serve as reliable predictors for isomerization energy trends in this class of molecules, offering valuable avenues for future exploration of their structural and energetic behaviors. As an example of how these parameters are used, the line of best fit from Equation (1)—minimized for the MAD using the C_36_H_20_ dataset and a combination of Σ_Dihedral_, HOMA, and *θ*_RMSD_—yields the following expression for the isomerization energy.

$$\Delta E_{\mathrm{iso}}=0.0207\;\Sigma_{\text{Dihedral}}-339.00\;\text{HOMA}+16.44\;\theta_{\text{RMSD}}+236.09$$

This is implemented directly into the PAH Automated Property Scanner, which also provides explicit equations upon use. The code for calculating each of the parameters is available on GitHub https://github.com/Kasigee/PAH_Analysis.

### 3.5 PAH Automated Property Scanner

We have developed an online tool, PAH Automated Property Scanner (PAHAPS, https://pahaps.streamlit.app/), for predicting isomerization energies from *xyz* structures by calculating the geometric properties or finding the isomer within this dataset via a SMILES matching algorithm. This is best used for C_16+4*n*_ H_10+2*n*_ PAHs, and it appears that HOMA and θ_RMSD_ can deviate significantly between XTB and DFT geometries, so we recommend using XTB‐optimized geometries (as this model was trained on these) or using “Dihedral‐only” (noting its limitations for planar PAHs). Relating to C_16+4*n*_ H_10+2*n*_ PAHs, parameters from different data subsets are most consistent, and the tool automatically calculates Σ_Dihedral_, *θ*_RMSD_, and HOMA values to estimate both isomerization energy and its error bounds (most reliable for similar polybenzenoid PAHs). Despite some lack of transferability to structurally diverse sets, these models remain faster and more accurate than unfitted XTB. They are also easily accessible, requiring no local installation or additional computations.

### 3.6 HOMA Analyses for XTB PAH Structures

We investigated whether the *R*_opt_ value used in Equation (1) could be optimized to enhance its utility as a metric for PAH isomerization energies. Figure S7 illustrates this analysis for HOMA used alone and in combination with the other parameters examined. For the full dataset (COMPAS‐3x’), there is minimal correlation between the chosen *R*_opt_ and the isomerization energies. Furthermore, the slopes (Figure 6b) and intercepts (Figure 7b) for HOMA vary considerably with chemical formula, indicating that HOMA is not readily transferable among different peri‐condensed systems. Indeed, the MADs of both isomerization energy and *R*_opt_ show unique trends for COMPAS‐3x’ compared to any single chemical formula, although within each chemical formula group, the trends are broadly similar but differ in magnitude.

Interestingly, for a given chemical formula, the lowest MAD occurs at *R*_opt_ values around 1.43–1.45 Å—significantly larger than the canonical 1.388 Å often cited for “ideal” aromatic bonds (Figure S7). Across our entire dataset, the average C–C bond lengths for each PAH range from 1.4037 to 1.4064 Å. However, within a single C_44_H_24_ structure, individual bond lengths can vary significantly—from 1.3605 to 1.4527 Å in the highest‐energy isomer and from 1.3507 to 1.4539 Å in the lowest‐energy isomer. The *R*_opt_ trends suggest that longer bonds may have less impact on overall stability than shorter ones. Although choosing *R*_opt_ values near the longest bonds did reduce the MAD for HOMA in isolation, it did not reduce the MAD when HOMA was paired with other parameters. In some cases, such as pairing with Σ_Dihedral_, it actually increased the MAD. Consequently, for the MAD data in Table 3 and Figures 4, 6, and 7, as well as the PAHAPS tool, we retained the 1.388 Å value to enable clearer comparisons with literature.

## 4 Conclusions

This study calculates the isomerization energies in the extensive COMPAS‐3x database of 39,482 PAH isomers, which have here been calculated at the PBE0‐D4/6‐31G(2df,p) level of theory, to identify structural parameters that predict relative stability. This computational approach builds on the high accuracy of PBE0‐D4, previously benchmarked against CCSD(T) reference values with a mean absolute deviation (MAD) of 0.67 kcal mol^−1^ for the PAH335 database [29], underscoring its reliability for studying large‐scale datasets. The use of this high‐fidelity dataset provides robust insights into the structural and energetic relationships within PAHs and sets a strong foundation for advancing stability predictions.

Our analysis highlights the importance of considering nonplanarity as a key structural factor affecting PAH stability, especially in larger PAHs where most isomers are nonplanar. Here we introduced the sum of dihedral deviations (Σ_Dihedral_), geometric predictors that demonstrated the strongest correlation with isomerization energies, achieving an MAD of 3.6 kcal mol^−1^ relative to the PBE0‐D4/6‐31G(2df,p) reference values. This parameter's global sensitivity to distributed distortions makes it superior to localized metrics like the maximum *z*‐displacement (Δ*z*), which has an MAD of 4.8 kcal mol^−1^. The HOMA parameter, despite its weaker standalone performance (MAD of 5.3 kcal mol^−1^), complements Σ_Dihedral_ by capturing planar aromaticity, and their combination reduces the MAD to 2.5 kcal mol^−1^. Further, but only subtle reductions (of less than 0.1 kcal mol^−1^ for the MAD) are observed by also including an angular parameter, the RMSD of angles away from the ideal 120°, *θ*_RMSD_. It appears that polybenzenoid PAHs of the form C_16+4*n*_H_10+2*n*_ have transferability across various system sizes for predicting isomerization energies. We provide an online tool, PAHAPS (https://pahaps.streamlit.app/), for predicting isomerization energies from xyz structures. Its best use case is for isomers already contained within the COMPAS3 or COMPAS3x’ datasets (via SMILES matching) or for PAHs of the form C_16+4*n*_H_10+2*n*_. Extending the model to other systems—such as PAHs with perfluorination [75], five‐membered rings, heteroatoms, sp^3^ centers—remains an open direction for future research.

Additionally, incorporating semiempirical GFN2‐xTB calculations with these geometric predictors further enhances the predictive accuracy. While XTB alone yields an MAD of 5.9 kcal/mol^−1^, fitting it to the dataset with a correction factor reduces this to 1.2 kcal/mol^−1^, and combining it with Σ_Dihedral_, *θ*_RMSD_ and HOMA achieves the lowest MAD of 0.8 kcal/mol^−1^. These results demonstrate the potential synergy between structural predictors and semiempirical methods, significantly improving computational efficiency while maintaining accuracy. However, more work is required to ensure a generalized model transferable to further datasets.

The findings underscore the utility of combining geometric and semiempirical methods for large‐scale stability predictions. Future studies could explore the application of these methods to catacondensed, functionalized, or non‐polybenzenoid PAHs, heteroaromatic systems, or machine learning frameworks to further reduce computational costs and broaden their applicability. By providing an accurate and scalable approach, this work contributes to the development of efficient tools for predicting PAH properties relevant to materials science and energy storage applications.

## Conflicts of Interest

The authors declare no conflicts of interest.

## Supporting information


**Data S1:** Supporting Information.

**Data S2:** Supplementary Information.

## Data Availability

The data that supports the findings of this study are available in the S1 material of this article.
