# Supplementary material for: Big‐Data Analysis of Geometric Descriptors as Efficient Predictors of Energetic Stability in Nonplanar Polycyclic Aromatic Hydrocarbons
Source: J Comput Chem. 2025 Aug 1;46(21):e70198. doi: 10.1002/jcc.70198 (PMC12315058; doi:10.1002/jcc.70198)
Supplement: Supplementary file 2 — Data S2: Supplementary Information. [file JCC-46-0-s002.docx]

**Big-Data Analysis of Geometric Descriptors as Efficient Predictors of Energetic Stability in Nonplanar Polycyclic Aromatic Hydrocarbons**

Kasimir P. Gregory^*^ and Amir Karton^*^

School of Science and Technology, University of New England, Armidale, NSW 2351, Australia

**Supporting Information**

**Table S2.** Error statistics with added correction factors (C) to the local XTB method and PBE0/6-31G(2df,p) for the planar and nonplanar isomers considered here relative to the PBE0-D4/6-31G(2df,p) isomerization energies (in kcal mol^–1^).*^a^*

| Subset | MAD | RMSD | Max Dev |
| --- | --- | --- | --- |
| C.XTB |  |  |  |
| Planar (1,978)*^b^* | 0.46 | 0.57 | 1.77 |
| Nonplanar (36,286)*^c^* | 1.16 | 1.54 | 10.11 |
| Everything (38,264) | 1.15 | 1.54 | 10.37 |
| C.PBE0/6-31G(2df,p) |  |  |  |
| Planar (1,978)*^b^* | 0.43 | 0.52 | 1.83 |
| Nonplanar (36,286)*^c^* | 1.41 | 2.18 | 17.59 |
| Everything (38,264) | 1.39 | 2.14 | 17.59 |

*^a^*MAD = mean absolute deviation, RMSD = Root-mean-square deviation, MSD = mean signed deviation, Max Dev = maximum deviation. *^b^*Planar or nearly planar isomers with ∆*z* values smaller or equal to 1.0 Å. *^c^*Nonplanar isomers with ∆*z* values larger than 1.0 Å.

**Discussion on the various HOMA value approaches**

Here, we have used various approaches to calculate the Harmonic Oscillator Model of Aromaticity (HOMA), each with varied success. The initial HOMA model we used, and indicated as HOMA within the main manuscript and Figures S1-S7, takes the HOMA of each ring j and then takes the average across all M rings:

$${\bar{\mathrm{HOMA}}}_{\mathrm{ring}}=\frac{1}{M}\sum_{j=1}^{M} \left( 1-\frac{\alpha}{n_{j}}\sum_{i=1}^{n_{j}} \left( R_{i,j}-R_{\mathrm{opt}} \right)^{2} \right)$$

Where j has n_j_ bonds of lengths $\{R_{j,1},\ldots,R_{j, n_{j}}\}$.However, this will inherently double-count contributions from fused bonds. If instead we take only the unique bonds from the set of bond B to obtain a global HOMA value via the following equation:

$$\mathrm{HOM}A_{\mathrm{global}}=1-\frac{\alpha}{N}\sum_{b\in B} \left( R_{b}-R_{\mathrm{opt}} \right)^{2}$$

However, this surprisingly performs worse than ${\bar{\mathrm{HOMA}}}_{\mathrm{ring}}$, perhaps indicating fused bonds have a higher influence on the aromaticity/ correlative power with this PAH database. So by looking only at the set of fused bonds, F, for the total N_f_ bonds:

$$\mathrm{HOM}A_{\mathrm{fused}}=1-\frac{\alpha}{N_{f}}\sum_{b\in F} \left( R_{b}-R_{\mathrm{opt}} \right)^{2}$$

Does indeed reveal an improved predictive performance over both $\mathrm{HOM}A_{\mathrm{global}}$ and ${\bar{\mathrm{HOMA}}}_{\mathrm{ring}}$. We also investigated just the set of edge bonds, E, for the total N_e_ bonds:

$$\mathrm{HOM}A_{\mathrm{edge}}=1-\frac{\alpha}{N_{e}}\sum_{b\in E} \left( R_{b}-R_{\mathrm{opt}} \right)^{2}$$

Which similarly outperforms $\mathrm{HOM}A_{\mathrm{global}}$. Σ_Dihedral_|$\mathrm{HOM}A_{\mathrm{fused}}$ obtained an MAD of 2.44 kcal mol^-1^ and Σ_Dihedral_|$\mathrm{HOM}A_{\mathrm{edge}}$ 3.05 kcal mol^-1^ compared to Σ_Dihedral_|$\mathrm{HOM}A_{\mathrm{global}}$ which has an MAD of 3.27 kcal mol^-1^. Σ_Dihedral_|${\bar{\mathrm{HOMA}}}_{\mathrm{ring}}$ had an MAD of 2.53 kcal mol^-1^.

We decided to also investigate a weighted-average HOMA, which can preserve the “local” ring definition of ${\bar{\mathrm{HOMA}}}_{\mathrm{ring}}$, but ensuring each bond’s total contribution across all rings is equivalent. For each bond b, we let $k_{b}$ = the number of rings it belongs to. Then for each ring j, define its weight:

$$w_{j}=\sum_{b\in ring j} \frac{1}{k_{b}}$$

Then,

$$\mathrm{HOM}A_{\mathrm{weighted}}=\frac{\sum_{j=1}^{M} w_{j}\left( 1-\frac{\alpha}{n_{j}}\sum_{i=1}^{n_{j}} \left( R_{i,j}-R_{\mathrm{opt}} \right)^{2} \right)}{\sum_{j=1}^{M} w_{j}}$$

Σ_Dihedral_|$\mathrm{HOM}A_{\mathrm{weighted}}$ obtained an MAD of 2.41 kcal mol^-1^. Considering $\mathrm{HOM}A_{\mathrm{fused}}$ is the simpler model for a similar error, we report these values over HOMA_weighted_ for clarity.


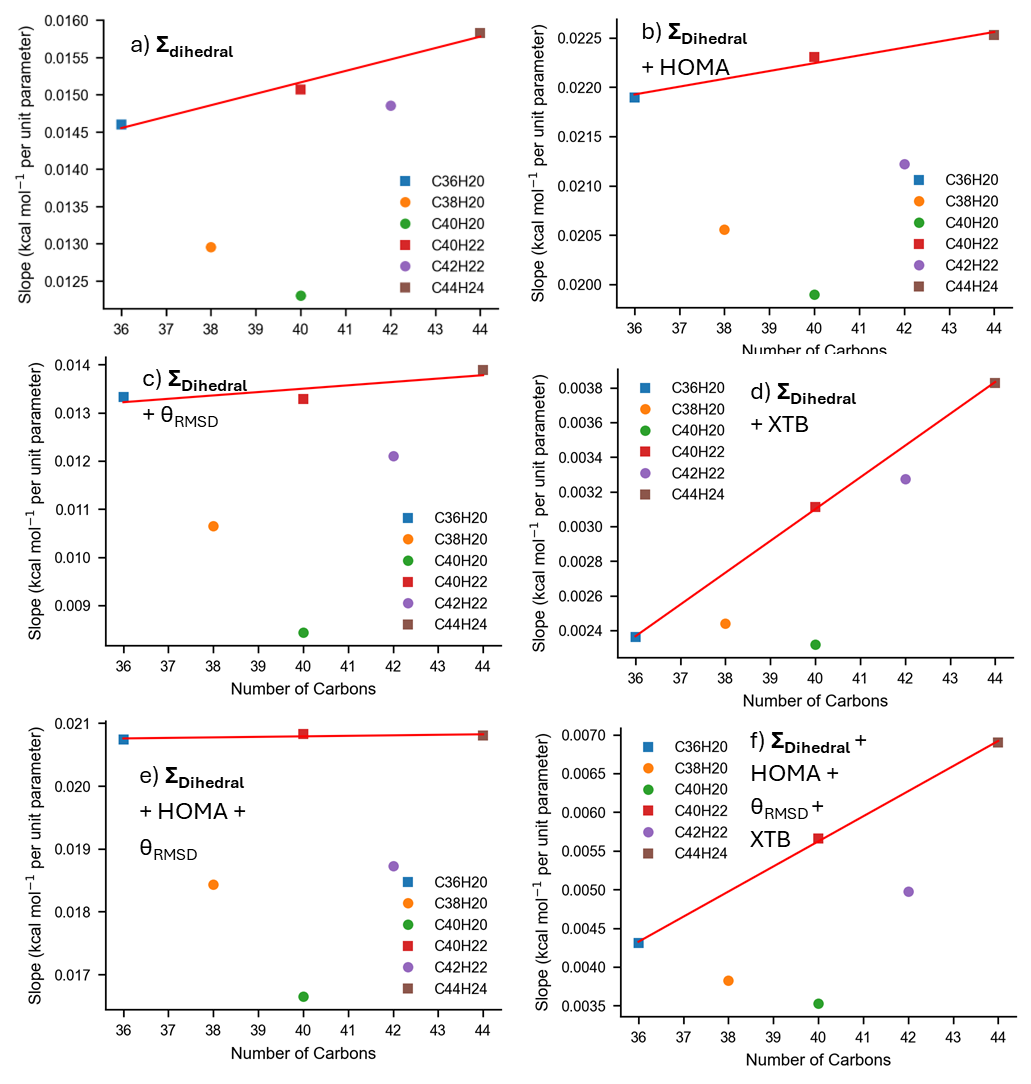


**Figure S1.** The effect of chemical formula slope relationship to the PBE0-D4/6-31G(2df,p) isomerization energies (in kcal mol^–1^) for Σ_Dihedral_ a) by itself, and when paired with b) HOMA, c) θ_RMSD_, d) XTB, e) HOMA and θ_RMSD_ and f) HOMA, θ_RMSD_ and XTB. Line of best fits shown for C_16+4n_H_10+2n_ (i.e., C_36_H_20_, C_40_H_22_, C_44_H_24_) structures which have a square symbol. Other chemical formulas have filled circles.


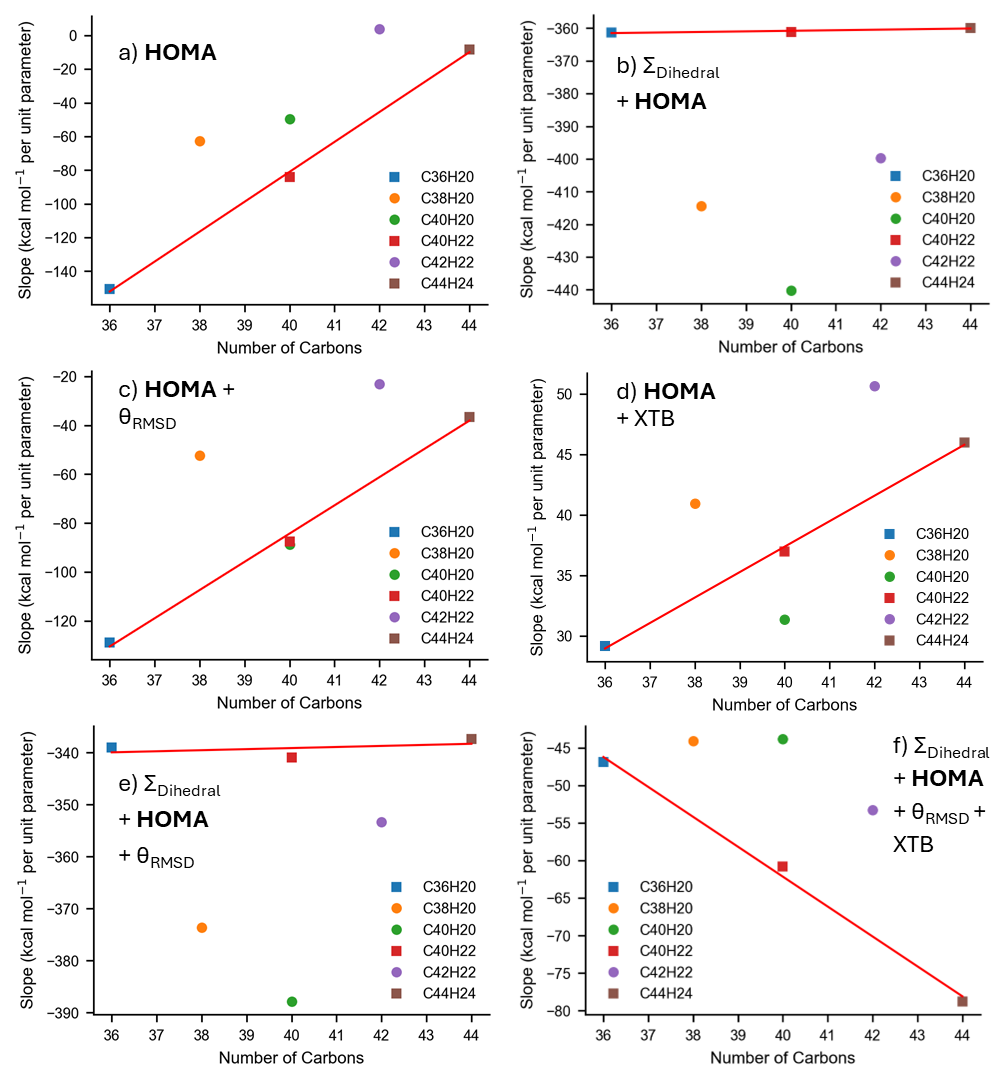


**Figure S2.** The effect of chemical formula slope relationship to the PBE0-D4/6-31G(2df,p) isomerization energies (in kcal mol^–1^) for HOMA a) by itself, and when paired with b) Σ_Dihedral_, c) θ_RMSD_, d) XTB, e) Σ_Dihedral_ and θ_RMSD_ and f) Σ_Dihedral_, θ_RMSD_ and XTB. Line of best fits shown for C_16+4n_H_10+2n_ (i.e., C_36_H_20_, C_40_H_22_, C_44_H_24_) structures which have a square symbol. Other chemical formulas have filled circles.


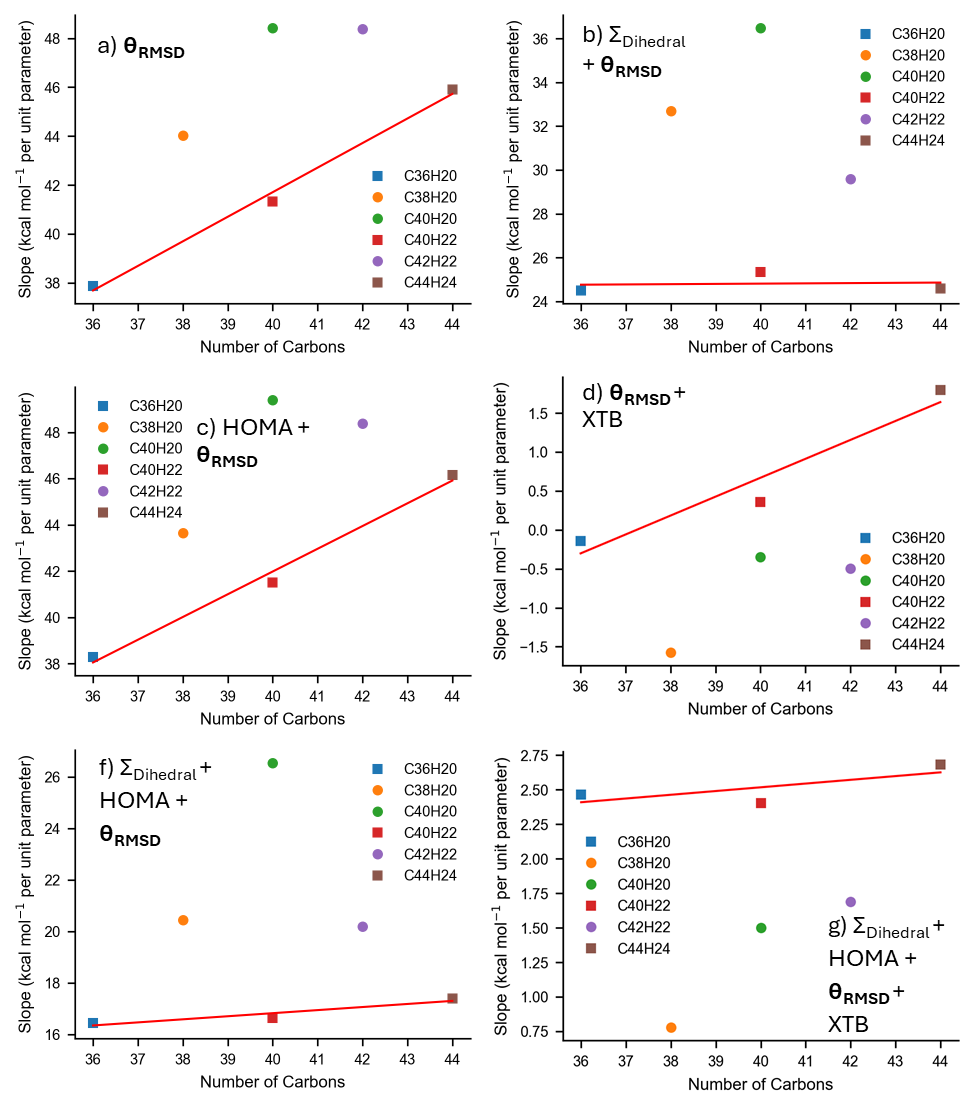


**Figure S3.** The effect of chemical formula slope relationship to the PBE0-D4/6-31G(2df,p) isomerization energies (in kcal mol^–1^) for θ_RMSD_ a) by itself, and when paired with b) Σ_Dihedral_, c) HOMA, d) XTB, e) Σ_Dihedral_ and HOMA and f) Σ_Dihedral_, HOMA and XTB. Line of best fits shown for C_16+4n_H_10+2n_ (i.e., C_36_H_20_, C_40_H_22_, C_44_H_24_) structures which have a square symbol. Other chemical formulas have filled circles.


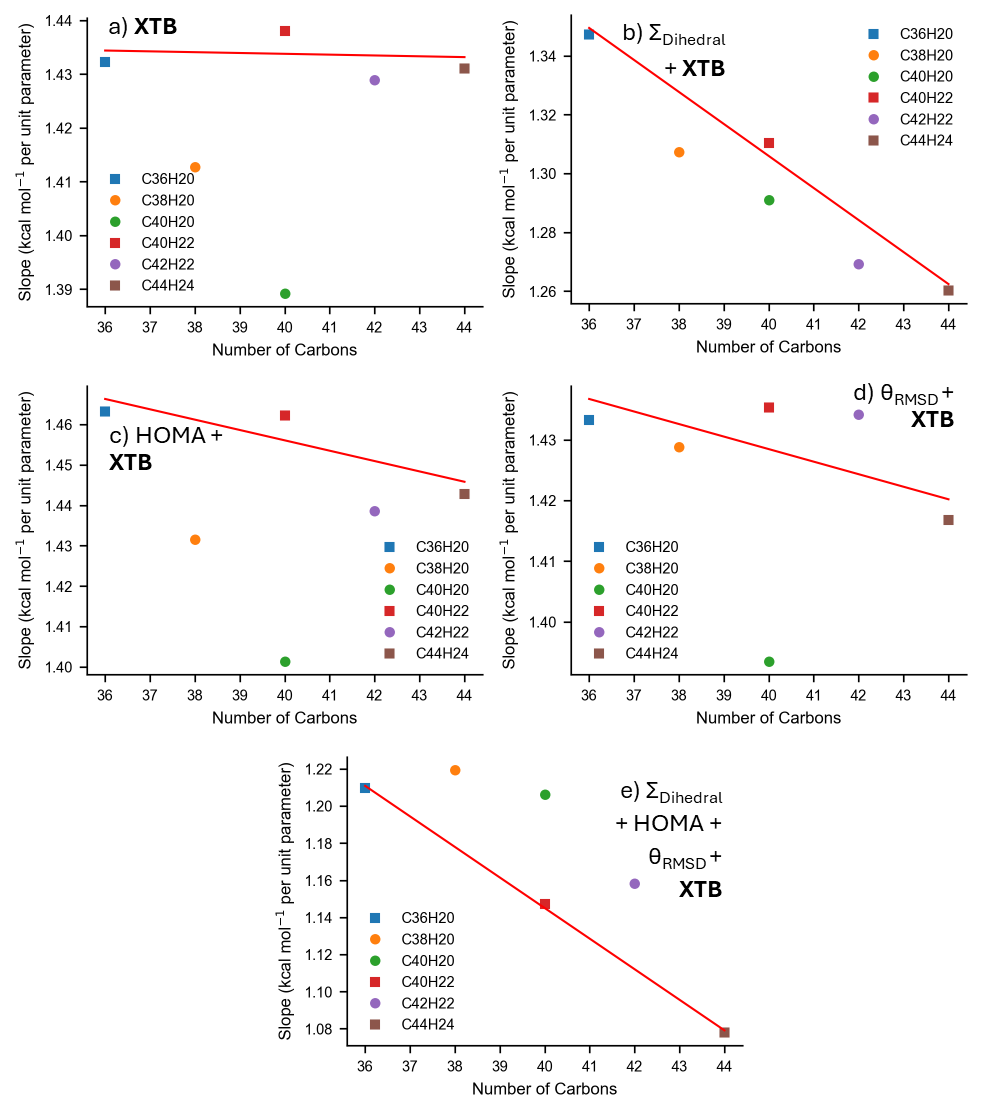


**Figure S4.** The effect of chemical formula slope relationship to the PBE0-D4/6-31G(2df,p) isomerization energies (in kcal mol^–1^) for XTB a) by itself, and when paired with b) Σ_Dihedral_, c) HOMA, d) θ_RMSD_, and e) Σ_Dihedral_, HOMA and θ_RMSD_. Line of best fits shown for C_16+4n_H_10+2n_ (i.e., C_36_H_20_, C_40_H_22_, C_44_H_24_) structures which have a square symbol. Other chemical formulas have filled circles. This reveals that XTB is most transferable in isolation, as compared to when combined with other parameters, albeit the Σ_Dihedral_ may have some utility if the trends continue, such that a size correction factor could be applied in these cases.

**
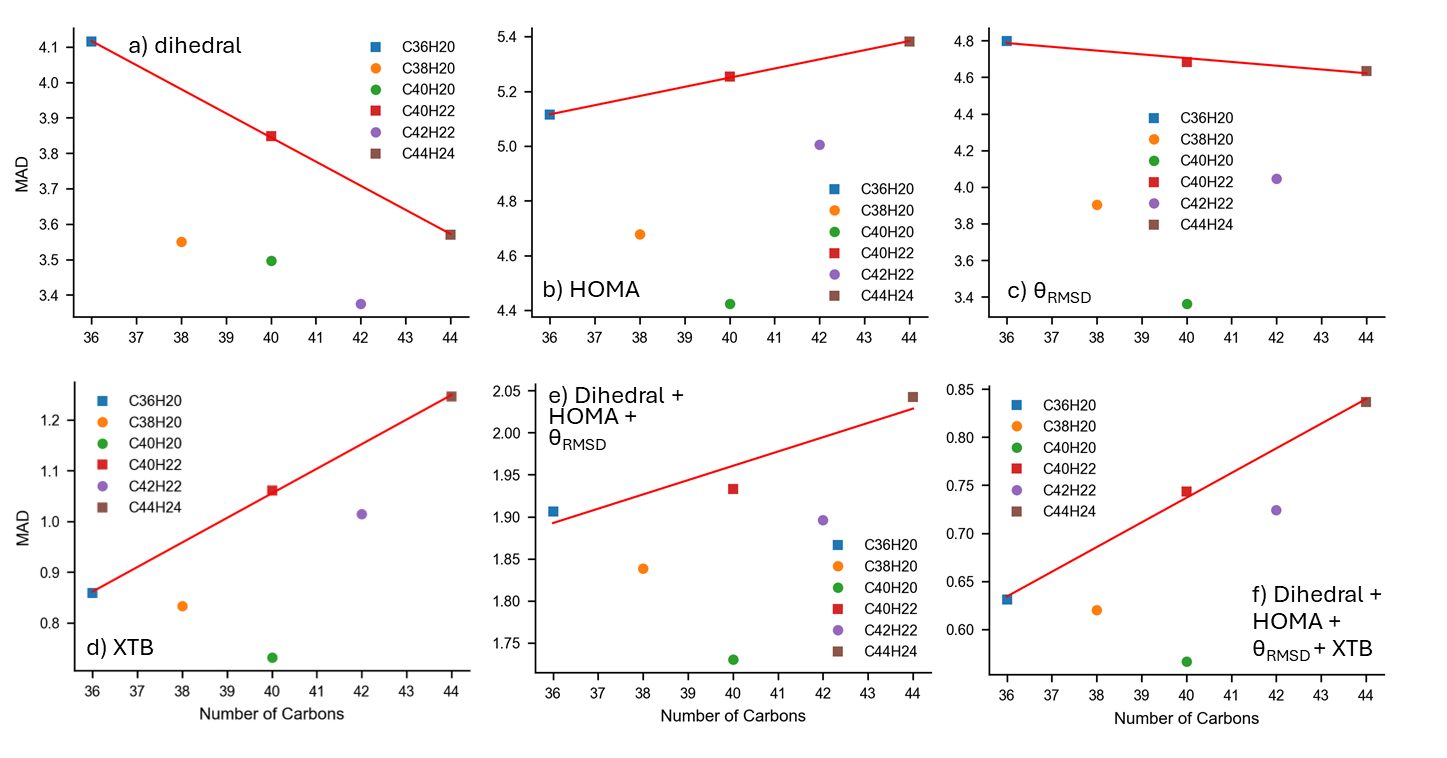
**

**Figure S5.** Plots of mean absolute deviation (MAD) from the PBE0-D4/6-31G(2df,p) reference isomerization energies (in kcal mol^–1^) versus the number of carbons for six different approaches: (a) Σ_Dihedral_, (b) HOMA, (c) θ_RMSD_, (d) XTB, (e) Σ_Dihedral_, HOMA and θ_RMSD_, and (f) Σ_Dihedral_, HOMA, θ_RMSD_ and XTB. Each marker represents a different chemical formula, with squares denoting the C_16+4n_H_10+2n_ series (i.e., C_36_H_20_, C_40_H_22_, C_44_H_24_) and circles denoting other formulas. Red lines show linear fits to the square-labeled data.


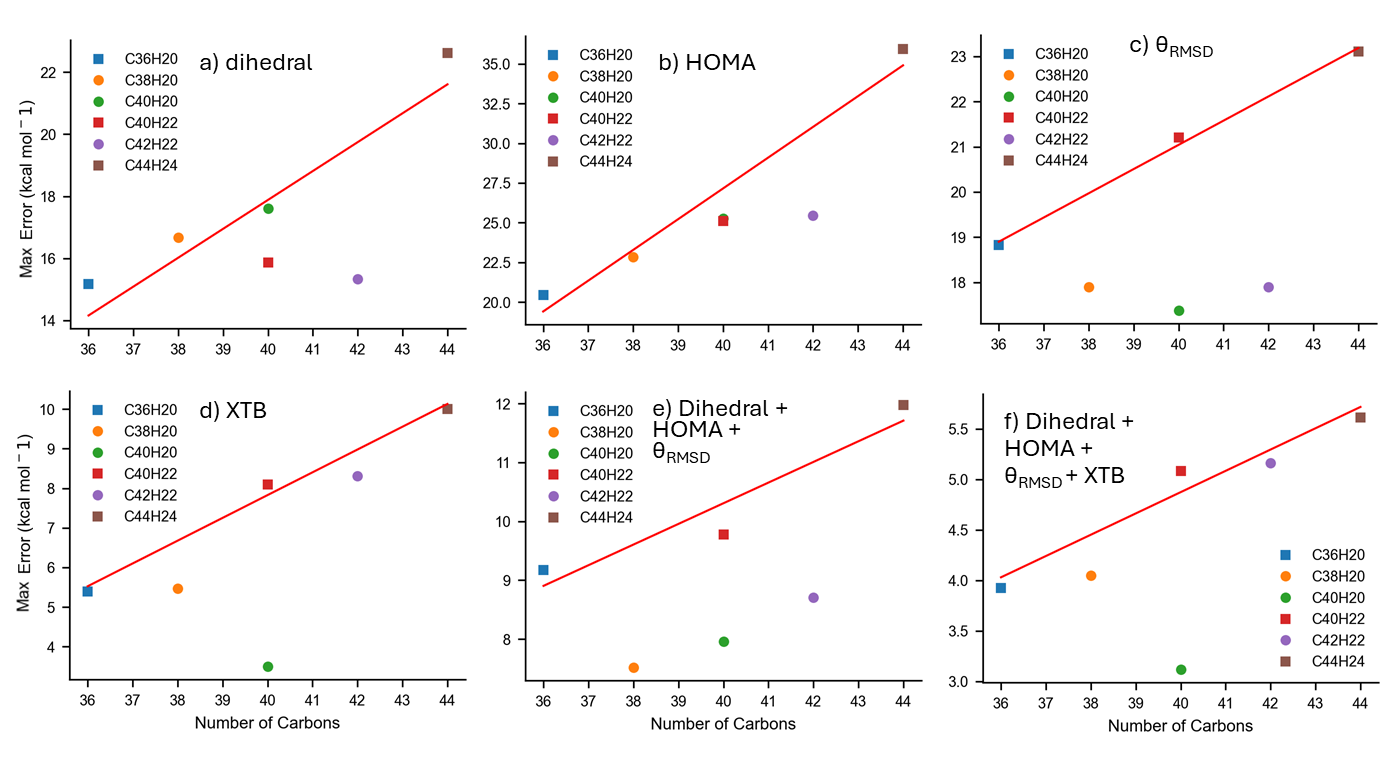


**Figure S6.** Plots of maximum error from the PBE0-D4/6-31G(2df,p) reference isomerization energies (in kcal mol^–1^) versus the number of carbons for six different approaches: (a) Σ_Dihedral_, (b) HOMA, (c) θ_RMSD_, (d) XTB, (e) Σ_Dihedral_, HOMA and θ_RMSD_, and (f) Σ_Dihedral_, HOMA, θ_RMSD_ and XTB. Each marker represents a different chemical formula, with squares denoting the C_16+4n_H_10+2n_ series (i.e., C_36_H_20_, C_40_H_22_, C_44_H_24_) and circles denoting other formulas. Red lines show linear fits to the square-labeled data.

**
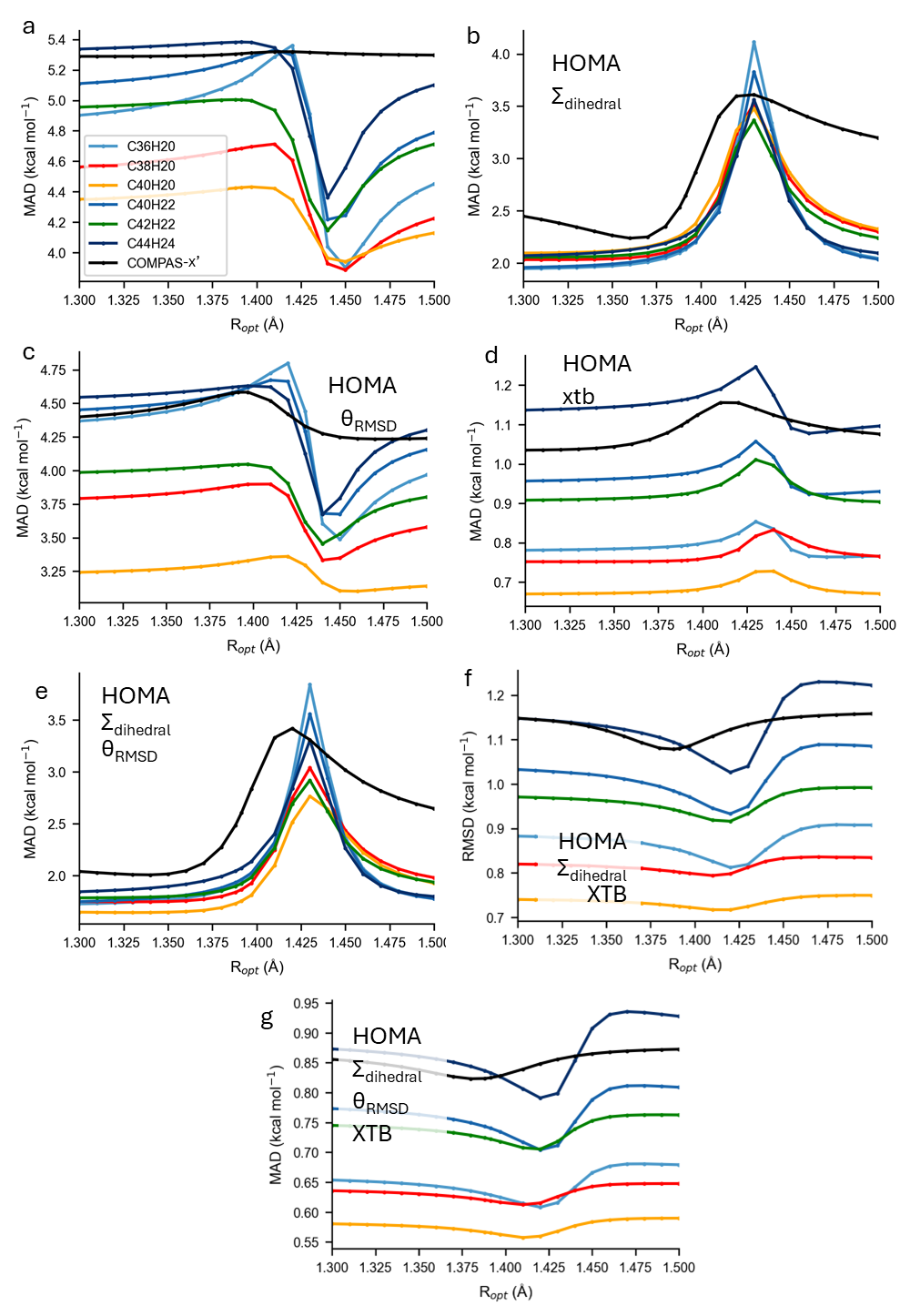
**

**Figure S7.** The effect of changing the R_opt_ value used in the HOMA calculation on the mean absolute deviation (MAD) of the fit between the PBE0-D4/6-31G(2df,p) reference isomerization energies (in kcal mol^–1^) versus the use R_opt_ value for the (a) lone HOMA calculation, as well as combined fits of HOMA with the (b) Σ_Dihedral_, (c) θ_RMSD_, (d) XTB, (e) Σ_Dihedral_ and θ_RMSD_, (f) Σ_Dihedral_ and XTB, and (g) Σ_Dihedral_, θ_RMSD_ and XTB. The C_16+4n_H_10+2n_ series, C_36_H_20_, C_40_H_22_, and C_44_H_24_ are in light, moderate and dark blue respectively.
